# Improving the quality of care for patients with hypertension in Moshupa District, Botswana: Quality improvement cycle

**DOI:** 10.4102/phcfm.v6i1.578

**Published:** 2014-04-25

**Authors:** Cathy Kande, Robert Mash

**Affiliations:** 1 Division of Family Medicine and Primary Care, Stellenbosch University, South Africa

## Abstract

**Background:**

Although there are no prevalence studies on hypertension in Botswana, this condition is thought to be common and the quality of care to be poor.

**Aim:**

The aim of this project was to assess and improve the quality of primary care for hypertension.

**Setting:**

Moshupa clinic and catchment area, Botswana.

**Methods:**

Quality improvement cycle.

**Results:**

Two hundred participants were included in the audit. Sixty-eight per cent were women with a mean age of 55 years. In the baseline audit none of the target standards were met. During the re-audit six months later, six out of nine structural target standards, five out of 11 process target standards and one out of two outcome target standards were achieved. Statistically-significant improvement in performance (*p* < 0.05) was shown in 10 criteria although the target standard was not always met. In the re-audit, the target of achieving blood pressure control (< 140/90) in 70% of patients was achieved.

**Conclusion:**

The quality of care for hypertension was suboptimal in our setting. Simple interventions were designed and implemented to improve the quality of care. These interventions led to significant improvement in structural and process criteria. A corresponding significant improvement in the control of blood pressure was also seen.

## Abstrait

**Améliorer la qualité de la prise en charge des patients souffrant d'hypertension dans le district de Moshupa, au Botswana: le cycle d'amélioration de la qualité**

**Présentation:**

Bien qu'il n'existe aucune étude portant sur la prévalence de l'hypertension au Botswana, il s'agit d'un problème de santé considéré comme courant, et la qualité de sa prise en charge est médiocre.

**Objectif:**

L'objectif de ce projet était d’évaluer et d'améliorer la qualité des soins de santé primaire liés à l'hypertension.

**Cadre:**

Centre médical et bassin de Moshupa, Botswana.

**Résultats:**

Deux cent personnes ont participé à l’étude. Soixante-huit pour cent d'entre elles étaient des femmes et l’âge moyen était de 55 ans. Au cours de l’étude subséquente menée six mois plus tard, six des neuf normes structurelles, cinq des 11 normes visées par le processus et une des deux normes visées par le résultat étaient effectives. Une amélioration statistiquement significative de la performance (*p* < 0.5) était observée pour 10 critères, bien que la norme visée n'ait pas été systématiquement atteinte. Au cours de l’étude subséquente, l'objectif consistant à procéder au contrôle de la tension artérielle (< 140/90) chez 70% des patients était réalisé.

**Conclusion:**

La qualité de la prise en charge de l'hypertension était inférieure à la norme dans notre cadre. Des interventions simples ont été élaborées et mises en oeuvres afin d'améliorer la qualité de cette prise en charge. Ces interventions ont conduit à une amélioration conséquente dans les critères structurels et relatifs aux processus. Une amélioration significative correspondante a également été observée dans le contrôle de la tension artérielle.

## Introduction

Although there are no prevalence studies on hypertension in Botswana, hypertension is thought to be very common.^1^ This condition is commonly asymptomatic, readily detectable by blood pressure measurement and can lead to complications if untreated.^2^ The extrapolated statistics for Botswana suggest that there may be at least 800 000 individuals with raised blood pressure, which represents approximately 41% of the total population.^3^ A cross-sectional study in Gaborone city council clinics in Botswana showed an even higher prevalence (61%) amongst the diabetic patients.^4^ Hypertension is the most common reason for outpatient medical review.^4, 5^

Hypertension has significant morbidity and mortality and is associated with adverse outcomes such as coronary artery disease, congestive cardiac failure, stroke and renal disease. This imposes more financial constraints on the health system, which is already burdened by the HIV pandemic. Based on the available evidence, the current US guidelines, published in the Seventh report of the Joint National Committee on prevention, detection, evaluation and treatment of high blood pressure (JNC 7), recommend maintaining blood pressure at less than 140/90 mmHg for most patients and less than 130/85 mmHg for patients with diabetes mellitus and renal disease.^5^ However, the Society for Endocrinology, Metabolism and Diabetes of South Africa recommend a target of 130/80 mmHg for type 2 diabetes.^6^

There is evidence that treatment to specific targets can reduce morbidity and mortality.^5, 7^ A reduction of 5–6 mmHg in diastolic blood pressure (DBP) has been shown to reduce the incidence of stroke by 40%, coronary events by 15% and heart failure by 50%.^5^ Non-pharmacological measures as well as medication can contribute to blood pressure reduction. For example, the Dietary Approaches to Stop Hypertension (DASH) low sodium diet reduces the systolic blood pressure (SBP) by 2–8 mmHg and weight reduction of 10 kg can contribute to a reduction of 5–20 mmHg.^5^

Despite this evidence, treatment in many settings is not very successful at achieving blood pressure control and meeting these targets. Control may remain poor as a result of limited resources, poor adherence to medication as well as inappropriate choice of medication. The National Health and Nutrition Examination Survey (NHANES III), conducted in the United States of America (USA) from 1988 to 1991, showed that 76% of known hypertensive patients had blood pressure measurements of 140/90 mmHg or higher.^5^ A study conducted on veterans in the USA from 1990 to 1995 showed that more than 65% had blood pressure measurements of 140/90 mmHg or higher and 40% had measurements of 160/90 mmHg or higher.^5^ Only 24% had their blood pressure controlled to less than 140/90 mmHg in a study conducted at five Department of Veterans Affairs sites in the USA.^8^

It is likely that the control of blood pressure and quality of care in Botswana is also problematic, although few studies exist to verify this. One study performed in Gaborone demonstrated that health workers frequently measure the patients’ blood pressure incorrectly.^4^

There are guidelines that have been developed locally to improve the care of hypertensive patients. The South African Hypertension Society guideline was published in 2011 and a Botswana guideline in 2007.^7, 9^ Both guidelines address the processes of care for hypertensive patients.

### Aim and objectives

The aim of this study was to improve the quality of care for hypertension at Moshupa clinic in Botswana. The specific objectives were to assess the current quality of care for hypertension; to plan and implement changes to improve the quality of care; to assess whether these changes were associated with a measurable improvement in the quality of care; and to make recommendations to the local Department of Health on how to improve the quality of care in primary care clinics.

## Research methods and design

### Ethical considerations

Ethical approval for the study was obtained from Stellenbosch University, reference N11/06/188.

### Study design

This project was a quality improvement cycle comprising the following steps:

- Establishing an audit team.
- Setting up of criteria and target standards.
- Data collection.
- Data analysis.
- Data interpretation.
- Planning of changes in the facility and the implementation of the changes.
- Re-audit to detect changes in the quality of care.^10^

### The setting

Moshupa district is situated in the southern part of Botswana. It has an estimated population of 22 811 which is served by 26 health facilities (eight clinics and 18 health posts).^11^ There is no hospital in Moshupa and the district relies on a mission hospital in Kanye for referral. Laboratory, radiograph facilities and ECG machines are non-existent in the district and all health facilities refer to the mission hospital for these investigations.

Clinics are staffed with an average of six health workers whilst health posts have an average of three health workers. Moshupa clinic (the main clinic in Moshupa) offers 24-hour services. It is staffed with 10 nurses working in shifts, a lay counsellor, one health auxiliary, two cleaners and two ambulance drivers.

There are six doctors in the district. They are involved in consultation of adults and children in primary care, maternity and emergency care. Each doctor is allocated a number of health facilities that they visit on a monthly basis, but they visit clinics more regularly than health posts.

A dedicated hypertension clinic does not exist in the district and hypertensive patients are seen daily, together with other general patients, by doctors and nurses with different levels of expertise. Antihypertensive drugs are refilled monthly at the local clinics. The following antihypertensive classes are available: thiazide diuretics, loop diuretics (furosemide), calcium-channel blockers (nifedipine), beta blockers (propranolol, atenolol), angiotensin-converting enzyme inhibitors (captopril, enalapril). Health education is organised in each health facility in the morning and covers a variety of topics, with talks delivered by the health workers. At times, patients are also asked to deliver a health talk under the supervision of a nurse or health education assistant.

The present study focused on the Moshupa council clinic and the three health posts that were in its catchment area: Lothlakane West health post, Moshupa health post and Ralekgetho health post.

### The audit team

The audit team was headed by the main researcher and included two doctors, one health auxiliary and four nurses. The team members felt motivated to participate in the quality improvement cycle as this would serve as a basis to audit other health programmes in which they had any active involvement. Prior to the audit, members had training on blood pressure measurement, which was not assessed in the study.

### Setting of criteria and target standards

The audit team opted to use the South African Society of Hypertension guidelines,^7^ which are the latest regional guideline based on internationally-accepted evidence. These guidelines also address the process of care in more detail and stratify the cardiovascular risk. The performance levels were set to be achievable targets and were based on the opinion of the audit team.

The South African Hypertension Society has established steps to follow to control BP. The first step is to evaluate the hypertensive patients with the following three objectives:^7^

1. to assess lifestyle and identify other cardiovascular risk factors or concomitant disorders that may affect prognosis and guide treatment
2. to reveal identifiable causes of high blood pressure
3. to assess the presence or absence of target organ damage and cardiovascular disease (CVD).

In a nutshell, lifestyle modifications reduce blood pressure, prevent or delay the incidence of hypertension, enhance antihypertensive drug efficacy and decrease cardiovascular risk.^5^ Further decisions with regard to the introduction of antihypertensive medications depend on the current blood pressure and the level of risk as described by the first step of evaluation of all hypertensive patients.

The choice of antihypertensive medications is also influenced by the presence of other co-morbid conditions, such as ischaemic heart disease, gout or asthma. In patients without such co-morbid conditions, the first antihypertensive medications are usually the thiazide or thiazide-like diuretics.

The guidelines classify hypertension as follows:

- Normal:
  - SBP 120–129 mmHg or DBP 80–84 mmHg
- High normal:
  - SBP 130–139 mmHg or DBP 85–89 mmHg
- Stage 1 (mild hypertension):
  - SBP 140–159 mmHg or DBP 90–99 mmHg
- Stage 2 (moderate hypertension):
  - SBP 160–179 mmHg or DBP 100–109 mmHg
- Stage3 (severe hypertension):
  - SBP >180 mmHg or DBP >110 mmHg

Furthermore, the guidelines recommend the following routine investigations:

- Body weight at every visit.
- Height at first visit.
- Body mass index at every visit.
- Abdominal obesity (waist circumference or waist-to-hip ratio) at every visit.
- Urinalysis at first visit and then yearly if normal. Repeated at the next visit if abnormal. If 2+ protein or 1+ haematuria, refer for or perform further investigations.
- Blood tests for creatinine, potassium, total cholesterol and fasting blood glucose should be done yearly.
- ECG should be done yearly.

Additional investigations are to be performed if secondary causes are suspected at the first visit or if refractory hypertension exists.

Treatment of hypertension should aim to achieve a target blood pressure of ≤140/90 mmHg for most patients; and a target blood pressure of ≤130/80 mmHg for patients with diabetes mellitus or chronic kidney disease.

The criteria were discussed during a meeting with the members of the audit team that agreed upon the performance levels. Performance levels were set for the structures relevant to hypertension management, the process of managing hypertensive patients and the outcome of the management of hypertension.

### Structure

One would expect certain items to be available at each facility. A score of two was assigned for full compliance, a score of one for partial compliance and zero for non-compliance. Partial compliance meant that items were present, but not in good working condition or in insufficient quantities or expired. Target standards for the structure were the presence of:

- one functional anaeroid sphygmomanometer (blood pressure machine) at each facility
- a small, medium and large blood pressure cuff at each facility
- one functional weighing scale at each facility
- one functional height scale at each facility
- the hypertension guidelines (2011 Southern African Hypertension Society) at each facility
- investigation request forms (laboratory and ECG) at each facility
- specimen tubes for blood tests at each facility
- an ECG machine at each facility
- specimen bottles for urine at each facility.

### Process

Target standards for the process were the following:

- 90% of records have the height recorded once
- 90% of records contain a weight measurement at each visit in the last year
- 90% of records have a classification of hypertension control in last year
- 70% of records demonstrate appropriate drug management as per the guidelines at each visit
- 60% of records have a serum creatinine recorded once yearly
- 60% of records have a fasting blood glucose recorded once yearly
- 60% of records have a record of urinalysis for protein, blood and glucose once yearly
- 90% of records have a random total cholesterol recorded once yearly
- 60% of records have an ECG recorded once yearly
- 80% of records have health education documented (either smoking, physical activity, diet or alcohol consumption) at each visit
- 90% of records have a body mass index recorded at each visit.

### Outcome

Target standards for outcomes were:

- 70% of records have a blood pressure < 140/90 mmHg
- 70% of records have a blood pressure < 130/80 mmHg for high risk patients.

### Study population

Our study population included all adult hypertensive patients aged 18 years and above, who visited the facilities with a documented diagnosis of hypertension over at least a six-month period. A sample-size calculation based on an 80% ability to detect a before–after difference with a *p* value of 0.05 recommended that 233 participants be included in the audit. Exclusion criteria were defined as pregnant women, patients from outside the Moshupa clinic catchment area and hypertensive patients aged less than 18 years.

### Data collection

Clients were selected systematically by taking every second patient with hypertension who walked into the consulting room for their usual review during November 2011. The folders were then put aside and retrospective data collected from the patient's file using a data collection tool in order to measure the defined criteria. This was done by the doctors and nurses in charge at each of the selected sites. The structural criteria were evaluated by an inspection of the facility by the audit team.

### Data analysis

Data were captured using a Microsoft Excel spread sheet and analysed by the Centre for Statistical Consultation at Stellenbosch University. Data analysis included frequency tables and comparison for significant change between the baseline audit and re-audit. Data were categorical in nature and a Chi-square test was used to detect significant differences (*p* < 0.05).

### Data interpretation

The results of the actual performance were presented to the audit team for discussion and comparison with the target standards.

### The planning and implementation of change

Plans for change in clinical practice were devised by the audit team to improve the quality of care of our hypertensive patients. The team had the mandate from the main researcher to ensure implementation and monitoring of any planned changes. A draft of the plan was submitted to every sister in charge and the management of the district.

### The re-audit

Data collection, data analysis and interpretation were repeated six months after the initial audit in June 2012. This period allowed time for the changes to be implemented. Further recommendations were formulated so as to improve the quality of care to an optimal level. The same hypertension tools and same standards were used during the re-audit.

## Results

### Patient characteristics

**FIGURE 1:**
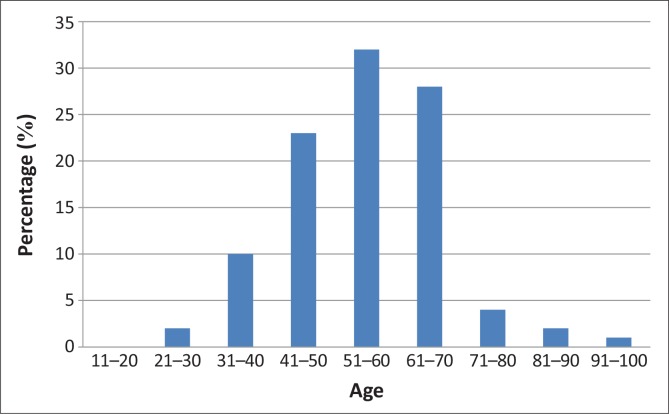
Age distribution of the sample.

There were 200 participants recruited from the four different sites: Moshupa clinic (*n* = 108; 54.0%), Moshupa health post (*n* = 56; 28%), Lotlhakane health post (*n* = 20; 10%) and Ralekgetho health post (*n* = 16; 8%). Moshupa had a larger population and the facilities there were busier than the smaller health posts. The participants’ mean age was 55 years and their age distribution is shown in Figure 1. Out of this sample, 68% were women and 32% were men.

### Structural standards

The performance levels for structural criteria in both audits are compared in Table 1. There were four clinics and a maximum score of eight for compliance with the structural standards. Table 1 shows that six standards were achieved at baseline and nine standards in the re-audit.

**TABLE 1:** Results for structural target standards.

Structure standards	Baseline audit (Maximum score = 8)		Re-audit (Maximum score = 8)	
	
	Score (%)	Standard achieved	Score (%)	Standard achieved
There is at least a weighing scale in each facility	8 (100)	Yes	8 (100)	Yes
There is at least a height scale in each facility	0 (0)	No	8 (100)	Yes
There are laboratory request forms in each facility	5 (63)	No	8 (100)	Yes
There are specimen bottles for blood collection in each facility	4 (50)	No	6 (75)	No
There are hypertension guidelines available in each facility	2 (25)	No	8 (100)	Yes
There are functional blood pressure machines in each facility	8 (100)	Yes	8 (100)	Yes
There is a small, medium, large cuff in each facility	8 (100)	Yes	8 (100)	Yes
There are specimen bottles for urine collection in each facility	4 (50)	No	6 (75)	No
There is a functional ECG machine in each facility	0 (0)	No	0 (0)	No

### Process standards

The performance level for process criteria are compared in Table 2 for both the baseline audit and the re-audit. Table 2 shows that none of the standards were achieved at baseline, but five out of 11 standards were achieved in the re-audit. There was a statistically-significant improvement during the re-audit for all the process criteria.

**TABLE 2:** Results for the process standards (*N* = 200).

Process standard	Baseline audit		Re-audit		
	
	*n* (%)	Standard achieved	*n* (%)	Standard achieved	*p*-value
90% of patients have control of hypertension classified	48 (24)	No	112 (56)	No	< 0.001
90% of patients have height measured	31 (15)	No	157 (79)	No	< 0.001
90% of patients have weight measured	144 (72)	No	167 (84)	No	0.002
90% of patients have BMI calculated at each visit	25 (12)	No	156 (78)	No	< 0.001
60% of patients have total cholesterol checked annually	51 (26)	No	137 (69)	Yes	< 0.001
60% of patients have fasting blood glucose checked annually	22 (11)	No	120 (60)	Yes	< 0.001
60% of patients have urine checked for protein, glucose and blood annually	19 (10)	No	120 (60)	Yes	< 0.001
60% of patients have ECG performed	44 (22)	No	122 (61)	Yes	< 0.001
60% of patients have creatinine checked once annually	44 (22)	No	117 (58)	No	< 0.001
80% of patients have health education documented at each visit	71 (35.5)	No	144 (72)	No	< 0.001
70% of patients have appropriate drug management at each visit	108 (54)	No	152 (76)	Yes	< 0.001

### Outcome standards

Table 3 shows that control of blood pressure improved significantly and that the target of 70% controlled with blood pressure below 140/90 mmHg was achieved, albeit narrowly, in the re-audit.

**TABLE 3:** Results for outcome standards.

Outcome standards	Baseline audit (*N* = 200)		Re- audit (*N* = 200)		
	
	*n*(%)	Standard achieved	*n*(%)	Standard achieved	*p*-value
70% of records with BP < 140/90	97 (49)	No	139 (70)	Yes	< 0.001
70% of record with BP < 130/80 for high risk patients	32 (16)	No	53 (27)	No	< 0.001

### Changes and implementation of changes

Recommendations were formulated by the audit team to address the poor performance seen in the baseline audit. Implementation of changes involved doctors, nurses and the pharmacist. The changes are summarised in Tables 4 and 5.

**TABLE 4:** Changes in clinical practice for structural standards.

Structure standards	Recommendation of audit team	Action taken
There is at least a weighing scale in each facility	Weighing scales should be ordered by the nurse in charge of the facility.	Audit meeting were held every Thursday. The nurse in charge at each facility checked daily to ensure that patients’ weight was taken at each visit.
There is at least a height measure in each facility	Height measure should be ordered by the nurse in charge.	Audit meeting were held every Thursday. Nurse in chargechecked daily to ensure that height was measured at least once.
There are specimen bottles for blood and urine collection	Each nurse in charge should order enough specimen bottles for his/her facility.	A physical count was made on a daily basis. An order was madeto the central medical store monthly or to the hospital for supply.
There are functional BP machines in each facility	Each nurse in charge should order BP machine for her/his facility.	An order of blood pressure machines was placed by the district pharmacist at the central medical store as part of her action plan.
There are hypertension guidelines in each facilityin the consulting room	Doctors should photocopy guidelines and make themavailable in each consulting room.	Guidelines were available in the consulting room for easy and quick reference. The guidelines were made available at each facility by the audit team.
There are request forms for investigations	Nurse in charge should order or photocopy enough request forms for his/her facility. Soft copiesshould be available on the main clinic computer.	Request forms were printed from the computer on a dailybasis and distributed to facilities where the audit was conducted. Forms were also ordered from central medical stores.

**TABLE 5:** Recommendations and actual changes for process standards.

Process standards	Recommendation of audit team	Action taken
90% of patients have their hypertension control assessed	Doctors and nurses to ensure that hypertension control is assessed for every patient.	An in-service training on hypertension guideline was held in clinics attended by nurses and doctors. Guidelines were distributed to participants. Meetings were held every Thursday afternoon. This needed to be reinforced by audit team.
90% of patients have their heightmeasured	Doctors and nurses to ensure that height is measured at first visit.	The health auxiliary officer at the screening point measured height for each patient height scale was checked every morning.
90% of patients have weight measured	Doctors and nurses to ensure that weight ismeasured at each visit.	The health auxiliary measured weight for each patient and checked the weight scale every morning.
90% of patients have BMI calculated	Doctors and nurses to ensure that BMI is calculated at each visit.	BMI calculation was taught during training. Calculation was done at screening point or in the consultation room.
60% of patients have cholesterol checkedand interpreted	Doctors and nurses to ensure that cholesterol is checked yearly.	Samples were collected from Monday to Thursday and transported to the hospital laboratory for analysis. The nurse in charge ensured specimens were transported in time. During our Thursday meeting emphasis was laid on the importance and interpretation of investigations in managing hypertensive patients.
60% of patients have fasting bloodglucose checked and interpreted	Doctors and nurses to ensure fasting blood glucose checked yearly.	Samples were collected and sent to hospital for analysis. An order for glucometers has been placed. Thursday meeting discussed interpretation of results.
60% of patients have urine checked for protein, glucose and blood	Doctors and nurses to ensure urine checked yearlyif normal and repeated at next visit if abnormal.	Samples were transported to the hospital from Monday to Thursday. Thursday meeting discussed urinalysis results.
60% of patients have ECG done and interpreted	Doctors to order ECG yearly.	There was a need to have an ECG machine locally. Clinics relied on the ECG machine at the hospital. Meetings were held to teach basic ECG interpretation. The audit team has advocated for purchase of at least one ECG machine in the district.
60% of patients have creatinine checkedand interpreted	Doctors and nurses to order creatinine yearly.	There was need to have laboratory facility in Moshupa. Meeting were held on Thursdays to review shortfalls identified by the audit.
80% of patients have health educationdone and documented	Doctors and nurses to ensure ongoing health education.	Needs to be reinforced. Counselling on life style modification – diet, exercise, smoking and alcohol consumption in the management plan. The points discussed were to be documented on the patient's card. Patients were issued with a written plan documented in the medical record and tailored to individual patients. Health educators assisted with morning health talks.
70% of patients have appropriate drug management	Doctors and nurses to ensure appropriate medication is prescribed.	Hypertension guidelines were made available in the consulting room. In service training assisted with adherence to the guidelines.

BMI, body mass index.

## Discussion

### Key findings and comparison with literature

The baseline audit demonstrated a poor quality of care for patients with hypertension and achieved none of the target standards. During the re-audit the structural criteria were the most improved, with six targets out of nine achieved. The process criteria showed five targets were achieved out of 11 and the outcome criteria met one target out of two. Significant improvement in performance was shown in 10 criteria although the target standard was not always met. Significant improvement in performance was the more important finding as the levels set were somewhat arbitrary and may have been too high and ambitious in many cases. It was possible to implement changes with simple interventions designed by the audit team and with regular follow up.

Improving the use of investigations was limited by the performance of the hospital-based laboratory, which often lacked reagents or had broken equipment. At the clinic level, the use of incorrect specimen bottles and forms resulted in some specimens being rejected.Our study showed improved blood pressure control with 70% of records recording a blood pressure of less than 140/80 mmHg in the re-audit. These results are supported by a number of other studies where improving the technical quality of care was an effective strategy for improving blood pressure control and was achieved by relatively simple interventions within quality improvement cycles.^12–15^

Furthermore, JNC7 suggests that healthcare providers give insufficient attention to health education.^5^ This study demonstrated that the frequency of health education improved, although the audit cannot assess the content or quality of the counselling.

Routine laboratory tests recommended in the South African guidelines included a 12-lead ECG, urinalysis, blood glucose, creatinine and total cholesterol. These laboratory tests were seldom carried out in the baseline audit, although this improved following the planning and implementation of change to clinical practice. This shows a lack of adherence on the part of the healthcare providers as suggested by the JNC 7 report.^5^ Primary care in Botswana has not been organised for the adequate management of non-communicable diseases, such as hypertension, and yet this audit shows that significant improvement can easily be made when attention is given to the requirements. At the end of the study, however, none of the clinics had easy access to an ECG machine.

### Limitations

Thirty-three patients were lost to follow up and the sample size with paired data was reduced to 200. The sample, however, appeared to be powered adequately to detect significant differences in the re-audit. Poor record keeping meant that not all activities were recorded in the notes and some patients kept their own records, which could not be traced at subsequent visits, resulting in missing data. The anaeroid sphygmomanometers were not calibrated regularly and thus the blood pressure measurements used in the audit were not taken under ideal conditions. The adherence of staff to the rules for blood pressure measurement was also not observed or assessed, although training on this was given prior to the audit.

### Recommendations and implications

Improvement in performance should be seen as an ongoing process so that improvements are maintained and further improvements targeted. Such a commitment must involve the local facility staff and the district management. Realistic performance targets should be set from the baseline findings and the audit extended to other facilities and health programmes run in the district.

The process criteria need additional interventions with regards to access to investigations and essential equipment. ECG machines, glucometers and urine dipsticks need to be procured. The district still relies on the mission hospital for most of the investigations. The audit team advocated for procurement of basic equipment for the main clinic as well as a laboratory facility for the district.

## Conclusions

The quality of care for hypertension was suboptimal in our setting as highlighted by the baseline audit. Simple interventions were designed and implemented to improve the quality of care of hypertensive patients. These interventions led to significant improvement in structural and process criteria. A corresponding significant improvement in the control of blood pressure was also seen. It is recommended that the quality improvement process be continued, expanded to other clinics and to other chronic conditions.
